# Crosstalk between dendritic cells and T lymphocytes during atherogenesis: Focus on antigen presentation and break of tolerance

**DOI:** 10.3389/fcvm.2022.934314

**Published:** 2022-07-28

**Authors:** Rossella Bellini, Fabrizia Bonacina, Giuseppe Danilo Norata

**Affiliations:** ^1^Department of Pharmacological and Biomolecular Sciences, University of Milan, Milan, Italy; ^2^Center for the Study of Atherosclerosis, E. Bassini Hospital, Cinisello Balsamo, Milan, Italy

**Keywords:** dendritic cells, T cell priming, break of tolerance, atherosclerosis, ApoB

## Abstract

Atherosclerosis is a chronic disease resulting from an impaired lipid and immune homeostasis, where the interaction between innate and adaptive immune cells leads to the promotion of atherosclerosis-associated immune-inflammatory response. Emerging evidence has suggested that this response presents similarities to the reactivity of effector immune cells toward self-epitopes, often as a consequence of a *break of tolerance*. In this context, dendritic cells, a heterogeneous population of antigen presenting cells, play a key role in instructing effector T cells to react against foreign antigens and T regulatory cells to maintain tolerance against self-antigens and/or to patrol for self-reactive effector T cells. Alterations in this delicate balance appears to contribute to atherogenesis. The aim of this review is to discuss different DC subsets, and their role in atherosclerosis as well as in T cell polarization. Moreover, we will discuss how loss of T cell tolerogenic phenotype participates to the immune-inflammatory response associated to atherosclerosis and how a better understanding of these mechanisms might result in designing immunomodulatory therapies targeting DC-T cell crosstalk for the treatment of atherosclerosis-related inflammation.

## Graphical Abstract

Atherosclerosis results from impaired lipid homeostasis and immune-inflammatory response, driven, among others, by the delicate balance between dendritic cells (DCs) and T lymphocytes. Physiologically, immature dendritic cells maintain immune homeostasis presenting self-antigen in a tolerant way, mainly influenced by the local milieu and by the antigen uptake itself. However, a chronic inflammatory condition promotes the maturation of DCs able to trigger the activation of T lymphocytes toward self-antigens, that appears to contribute to atherogenesis. 
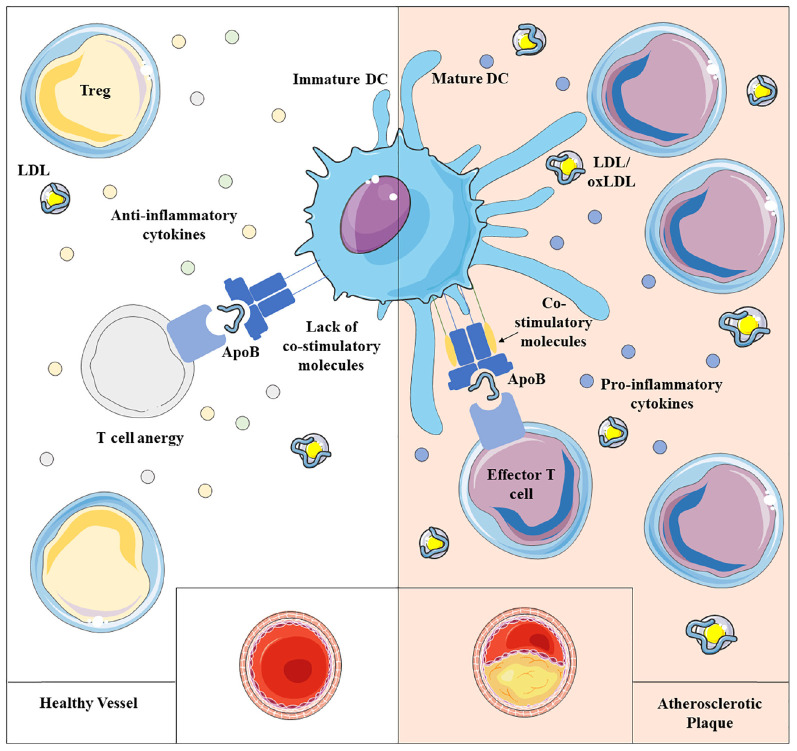

## Introduction

Lipid deposition in the arterial wall promotes the activation of an immune-inflammatory response which contributes to the development and progression of vascular lesions. Some evidence from experimental and clinical research has demonstrated that, during atherogenesis, an adaptive immune response mounts against naïve and modified epitopes of apolipoprotein B (ApoB), the main protein of low-density lipoproteins (LDL), and/or against oxidized lipids carried by LDL. The generation of T cells which become reactive against “self” and “modified self” antigens supports the hypothesis that a “break-of-tolerance” toward ApoB-containing lipoproteins might contribute to atherogenesis (1).

ApoB plays a critical role in the assembly of lipoproteins (chylomicrons and very low-density lipoproteins -VLDL-) and allows lipoprotein uptake by the LDL receptor (LDLR). ApoB, however, interacts also with proteoglycans (PGs) present in the arterial wall thus leading to lipoprotein retention (2). Retained lipoproteins promote atherogenesis by inducing the activation of an immune-inflammatory response in the arterial wall. Interestingly, although retained lipoproteins could undergo oxidative modification - leading to their conversion into oxidized LDL (oxLDL), that are recognized as a “not-self” moieties -, the possibility that a break of immune tolerance mounts against naïve LDL components contributing to atherogenesis is gaining a lot of attention (3).

Both these scenarios implicate that ApoB epitopes in their naïve or modified form are recognized by specialized antigen presenting cells (APCs), processed and presented to T cells which are not eliminated by mechanisms of immune tolerance, but rather promote an inflammatory response (4).

Indeed, although T cell polarization gives the final imprint to the immune response associated to atherosclerosis, APCs—that intercede in this process—play a relevant role. Macrophages and DCs are both considered APCs thanks to their ability to present antigens via major histocompatibility complexes (5). The role of macrophages has been deeply studied over the years, highlighting their role as prominent inflammatory cells, specialized in clearing necrotic and apoptotic material through phagocytosis (6) and also involved in polarizing adaptive response (7). More recently, the evolving techniques for cells identification and characterization have deepen our understanding of immune cells composition within the atherosclerotic plaque, showing that parallel to macrophages, other APCs, as dendritic cells (DCs), could participate to shape the polarization of atherosclerosis-related immune response (8).

Aim of this review is to discuss the role of DC and T cell crosstalk and its contribution to the break of tolerance toward ApoB during atherogenesis.

## Dendritic cell subsets in the context of atherosclerosis

Dendritic cells (DCs) are professional antigen presenting cells (APCs) devoted to the processing and presentation of specific antigens to T cells, both of self and non-self origin. The encounter with non-self antigens promotes the migration of immature DCs, that patrol tissues, to lymph nodes where they mature to competent APCs, thus supporting T cell activation and differentiation through the expression of co-stimulatory molecules and the production of pro-inflammatory cytokines. Vice versa, the presentation of endogenous antigens results in peripheral tolerance through the expression of co-inhibitory molecules on T cells paralleled to the production of inhibitory stimuli by DCs. The impairment of these mechanisms during antigen presentation results in either hyperactivated or hypo/non-responsive T cells, leading to the impairment of self-tolerance (9).

However, this fine balance can be impaired by other factors, as cholesterol levels that have a double-sward effect. Indeed, on one hand, APCs requires cholesterol for the correct internalization of antigens through micropinocytosis (10), but on the other, altered cholesterol efflux pathways and cholesterol cellular accumulation increase DCs function by affecting the composition of lipid rafts and the clustering of major histocompatibility complex class II (MHC-II) in these domains that is necessary for antigen presentation (11–13). In addition to this, the intracellular accumulation of cholesterol can modulate factors involved in APC proliferation and survival (14) and high cholesterol levels can lead to antigen modifications which enhance the immune response against modified antigens (10). Altogether these highlight the role of cholesterol levels in the modulation of antigen recognition and presentation of APCs (10).

In the context of vascular biology, tissue resident DCs normally patrol the intima layer of healthy aorta (15, 16) cooperating in preserving arterial homeostasis, while during atherogenesis, activated DCs infiltrate atheroprone regions in experimental models (15, 17) and in humans (18). Antigens related to atherosclerosis, such as native or modified ApoB, can be sensed by circulating, peripheral and arterial-infiltrated DCs, which then migrate toward para-aortic lymph nodes (19, 20) where antigens can be presented to naïve T cells. Antigen uptake is also mediated by scavenger receptors, among others the macrophage receptor with collagenous structure (MARCO), that plays a role in mediating intracellular signaling and Toll-like receptor (TLR) activation, including the modulation of DC morphology and migration (21–26). Indeed, their detection within the aortic plaque has recently suggested that DCs can migrate to the intima of the arterial wall where antigen can be presented, contributing to local activation of T cell or memory T cell restimulation (19, 20).

**FIGURE 1:**
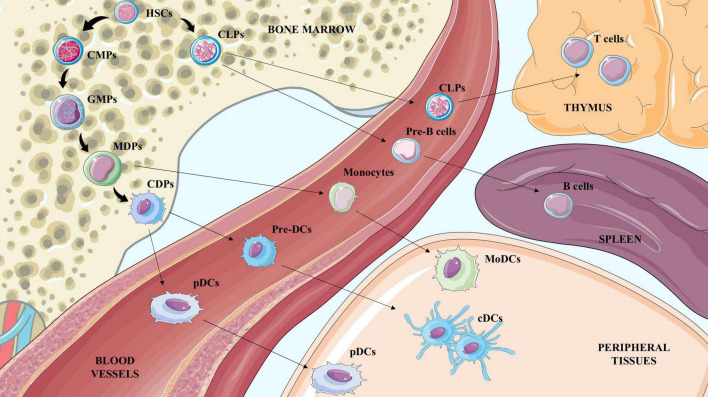
Illustration of hematopoietic stem cells (HSCs) differentiation to terminal blood and tissue cells. Hematopoietic stem cells (HSCs) generate common lymphoid progenitors (CLPs) and common myeloid progenitors (CMPs). CLPs exit the bone marrow to reach the thymus or the spleen and generate lymphocyte T and lymphocyte B cells respectively. CMPs differentiate to Granulocyte-Macrophage Progenitors (GMPs) which are the precursors of monocyte-DC progenitors (MDPs), and common DC progenitors (CDPs). CDPs mature to preDCs and pDCs in the bloodstream; once in the tissue preDCs differentiate in conventional DCs (cDCs). MDPs mature to monocytes in the blood and once in peripheral tissues give rise to macrophages and also to MoDCs.

Although belonging to APCs, DCs are a cluster of different cell subsets with a specific origin, role and function during the immune response. They are usually classified according to the expression of specific markers in conventional dendritic cells (cDCs), further divided in conventional type 1 or 2, plasmacytoid dendritic cells (pDCs) and monocyte-derived dendritic cells (MoDCs). While cDCs mainly instruct lymphocytes, pDCs are responsible for interferon (IFN) secretion (27) and MoDCs constitute a more heterogeneous subset which exerts both cDC-like function related to antigen presentation but also possesses macrophage features such as the capability of pathogen killing and phagocytosis (28). Similar to monocytes, DCs originate in the bone marrow from the hematopoietic common myeloid progenitors (CMPs) and granulocyte-monocyte progenitors (GMPs), which differentiate first in macrophage-dendritic cell progenitors (MDPs) and later to monocytes or MoDCs, or in common dendritic cell progenitors (CDPs), an exclusive DC precursor subset. CDPs then give rise to pDCs, which exit the bone marrow and circulate in the blood stream, or pre-DCs, which develop into cDCs in peripheral tissues, both lymphoid and not (29, 30) (Figure 1). The contribution of DCs during atherosclerosis is the result of the balance between the activity of the different subsets that could either promote the activation or the resolution of the immunoinflammatory response. Each DC subset presents a specific marker profile (Table 1) and can be detected in healthy as well as in atherosclerotic vessels. As a point of note, methodologies to investigate the role of DCs and their subsets are still controversial because both the use of full knockout mice or CD11c-depletion approaches by CD11c.DTR and CD11c.DOG models are not sufficient for deplete selectively DCs (31, 32), as other immune subsets could be affected thus contributing to the observed phenotype.

**TABLE 1:** Key markers of DCs subsets in lymphoid and non-lymphoid tissues.

	cDC1	cDC2	pDCs	MoDCs
Lymphoid tissues	CD11c^+^, CD8^+^, XCR1^+^	CD11c^+^, CD4^+^	CD11c*^int^*, Siglec H^+^, B220^+^, PDCA-1^+^, Ly6C^+^	CD11c^±^, MHCII^±^, CD11b^+^, CD64^+^ CX_3_CR1^+^, DC-SIGN^+^
Non-lymphoid tissues	CD11c^+^, MHC II^+^, Clec9A^+^, DEC205^+^, CD103^+^	CD11c^+^, MHCII^+^, CD11b^+^, CD172a^+^ CX_3_CR1^+^		

### Conventional type 1 dendritic cells

Normally, type 1 conventional dendritic cells (cDC1)—(classified as CD8^+^ in lymphoid tissues or CD103^+^ in non-lymphoid tissues) participate to the activation of the cytotoxic response of CD8^+^ T cells which results from the activation of the interferon regulatory factor 8 (IRF8) (33), of the basic leucine zipper transcription factor ATF-like 3 (BATF3) (34), of inhibitor of DNA binding 2 (ID2) (35) and of the FMS-like tyrosine kinase 3 (FLT3) (36). The contribution of cDC1 to atherosclerosis has been studied by profiling atherogenesis in experimental models lacking the expression of these factors (Table 2). In general, cDC1 depletion approaches in experimental models of atherosclerosis were associated with decreased atherogenesis. Indeed, DCs-restricted *Irf8* deletion—mainly affecting CD103^+^ cDC1—was associated with reduced T cell infiltration and atherosclerotic lesion development (37) and, similarly, *Batf3* depletion, leading to a significant decrease of CD103^+^ DCs within the aorta, reduced Th1 polarization and IFN-γ production, thus protecting from atherosclerosis (38). In parallel, the deficiency of Clec9a/DNGR1, a specific cDC1 receptor involved in sensing and presentation of necrotic cells-derived antigens, resulted in a lower number of macrophages and T cells infiltrating the atherosclerotic lesions, and reduced atherosclerosis in *Ldlr^–/–^* mice (39). Despite the consensus that cDC1 promote atherogenesis, still not all reports are concordant; this is the case of FLT3 deficient mice on *Ldlr^–/–^* background that—despite presenting less cDCs—were characterized by increased lesion size, together with increased levels of inflammatory cytokines (IFN-γ and TNF-α). However, as a reduction of immunosuppressive regulatory T cells (Tregs) was reported in this model, the possibility that FLT3-cDC1 mediates Tregs induction and this, in turn, modulates atherogenesis could not be excluded (36). Indeed, the lack of other cytokines produced by CD103^+^ DCs, such as TGF-β and/or retinoic acid that sustain Treg polarization and/or activation, could contribute to explain the unexpected results in these experimental models (40).

**TABLE 2:** Overview of studies on DC subsets and atherosclerosis-related phenotype.

	Atherosclerosis mouse model	Mouse models of DC deficiency	Effects on immune response	Effects on atherosclerosis
cDC1	*Ldlr^–/–^*	*Irf8^–/–^*	Lymphoid CD8C and non-lymphoid CD103C DCs ablation Decrease T cell levels	Atheroprotective (37)
		*Flt3^–/–^*	Decrease Tregs levels Increase inflammatory cytokines	Proatherogenic (36)
	Irradiated *Ldlr^–/–^*	Cd11c*^Cre+/^*Irf8*^flox/flox^* transplantation + Clec9a^–/–^ BM	Decrease plaque macrophages Decrease plaque T cells	Atheroprotective (39)
	*Apoe^–/–^*	*Batf3^–/–^*	Decrease splenic CD8^+^ DCs Decrease aortic CD103^+^ DCs Decrease Th1 levels Decrease IFN-γ production	Atheroprotective (38)
cDC2	Irradiated *Ldlr^–/–^*	CD11c*^cre+/^*Atg16l1*^flox/flox^* BM (autophagy)	Increase aortic Tregs Decrease foam cell formation	Atheroprotective (43)
	*Apoe*-^–/–^	CCL17*^E/E^* (chemokine)	Decrease Tregs levels Increase T effector polarization	Proatherogenic (44)
pDCs	*Apoe^–/–^*	CpGs injection + anti-mPDCA-1 Ab injection	Decrease IFN-α production	Atheroprotective (46)
		anti-mPDCA-1 Ab injection	Aortic and splenic pDCs depletion	Atheroprotective (47)
	*Ldlr^–/–^*	*CD11c-Cre - Tcf4^–/flox^* BM	Decrease T cells lesional infiltration	Atheroprotective (48)
		PDCA-1 depletion	Increase plaque T cells	Proatherogenic (49)
		BDCA2-DTR BM	Decrease lymphoid pDC levels Decrease aortic Tregs	Proatherogenic (50)
		*Ido1^–/–^*	Decrease aortic Tregs	Proatherogenic (50)
MoDCs	*Apoe^–/–^*	*CX_3_CR1^–/–^*	Decrease intimal DCs	Atheroprotective (56)
	*Ldlr^–/–^*	*GM-CSF^–/–^*	Decrease lesional DCs Decrease CD4^+^ T helper levels Decrease CD8^+^ cytotoxic T cell levels	Atheroprotective (57)

Whether these contrasting findings reflect a plastic role of cDC1 on atherogenesis related to a specific condition influencing their activation still remains to be addressed.

### Conventional type 2 dendritic cells

The type 2 conventional dendritic cells (cDC2) generally promotes Th2, Th17 or Treg responses (41). Within the intima of the aorta, cDC2 represent the most abundant resident DC subset and increases during atherogenesis (42). According to the heterogeneity of cDC2, also referred as CD11b^+^ cDCs, approaches to investigate their role in atherogenesis were focused on addressing the impact of changes in cDC2 specific functions, such as autophagy. For example, DCs lacking ATG16L1 (a key protein for autophagy) in *Ldlr^–/–^* mice presented a more tolerogenic phenotype associated with the expansion of Tregs and the reduction of effector T lymphocytes and of Th1-related cytokine production (43). It is worth noting that this “atheroprotective effect” was strictly related to cDC2 (43). On the other hand, hypercholesterolemic *Apoe^–/–^* mice showed an inverse correlation between the extension of aortic lesion and the number of circulating CD11b^+^ cDCs, paralleled by higher expression of chemokines receptors -such as CCR5 and CCR7- which cast for a more intense trafficking of cDC2 to the atherosclerotic plaque (42). In line with this, the CCL17 (or thymus and activation regulated chemokine -TARC-) exclusively produced by CD11b^+^ cDCs, has been proven to strongly promote inflammatory T cells while suppressing Treg response (44), thus suggesting a proatherogenic function of cDC2.

These findings suggest that, even in the context of cDC2, it is difficult to define an “*a priori*” role of this subset in atherosclerosis which rather could be influenced by local signals produced during atherogenesis.

### Plasmacytoid dendritic cells

Plasmacytoid dendritic cells (pDCs) represent a peculiar subset of DCs, which were shown to colocalize with T cells in the atherosclerotic plaque (18), suggesting that a functional contact *in situ* exists (45). pDCs are the first line of defense against viruses and bacteria as they are able to recognize nucleic acids, probably derived from dying cells, as well as immune complexes, thus promoting IFN-α production which has been associated to atherogenesis (45). In the context of atherosclerosis, pDCs are activated by immune complexes containing self-DNA from dying cells in the plaque and fuel atherosclerosis-associated immune response against self-molecules thus contributing to disease progression (46). pDC deficiency in *Apoe^–/–^* mice induced by anti–mPDCA-1 (anti mouse-plasmacytoid dendritic cell antigen-1) treatment, an approach broadly used to deplete specifically pDCs (47), protects from atherosclerosis (46, 47), either by direct reduction of IFN-α production (46), or indirectly by reducing Th1 proinflammatory cytokines and chemokines, such as IL-12, IFN-γ and CXCL1, CXCL10 (47). pDC development was also blocked by CD11c-restricted deletion of *Tcf4*; intriguingly when the bone marrow of these mice was transplanted into irradiated *Ldlr^–/–^* mice, fed on high-fed diet (HFD) a reduced atherosclerotic plaque area with lower T cells accumulation was observed (48).

Although these studies support a proatherogenic role of pDCs, other studies testing strategies blocking specific pDC markers, such as the inhibition of PDCA-1 or BDCA2 in *Ldlr^–/–^* mice, reported a worsening of the atherosclerosis, with plaques characterized by a more unstable phenotype and increased T cell accumulation (49, 50). This response has been proposed to depend on reduced expression of 2,3-dioxygenase 1 (IDO-1), a tolerogenic enzyme critical to drive the generation of Tregs by pDCs; indeed, *Ido1* deficiency in atheroprone *Ldlr^–/–^* mice was associated with an increased susceptibility to atherosclerosis development (50).

Albeit these studies showed that pDCs are mostly atherogenic, some specific molecules produced by pDCs appear to play an atheroprotective role. As such, further studies are necessary to clarify the role of this DC subset during atherosclerosis.

### Monocyte-derived dendritic cells

The expression of both DC and macrophage markers, such as MHC II, CD11c and F4/80, CD64 respectively is peculiar of MoDCs (28). The transition from monocytes to MoDCs usually takes place during inflammation, thus highlighting the role of this subset as antigen presenting cells within lymph nodes, rather than preserving tissue homeostasis in steady state conditions (51). MoDCs are an heterogenous population of DCs that are identified, similar to cDCs, by the expression of Zbtb46, CD103 or high levels of CD11b—among the most known (29). Classical monocytes give rise to macrophages or MoDCs in the presence of colony-stimulating cytokines such as M-CSF and GM-CSF respectively (52, 53), while non-classical ones mainly patrol non-inflamed tissues and depend on CX_3_CR1 recruitment (29). However, some evidence suggests that CX_3_CR1 is as well implicated in atherosclerosis by recruiting monocytes into the lesion, as recently confirmed by *Cx_3_cr1* depletion which impairs DC as well as macrophage accumulation in aortic lesion, thus resulting in reduced atherosclerotic plaque (54–56). In parallel, the proatherogenic function of monocyte-derived cells was further confirmed in CX_3_CR1 or GM-CSF deficient mice crossed with atheroprone *ApoE^–/–^* and *Ldlr^–/–^* mice, despite it should be noted that these molecules are not specific only for DCs (56–58), but they also promote the migration, adhesion and proliferation as well as the survival of natural killer cells, T cells, and smooth muscle cells (59).

Among different DC subsets, MoDCs engulf lipids, including aggregated LDL (agLDL), thus becoming foam cells (60). MoDCs localize in the atherosclerotic vessel—due to impaired migration—thus increasing the chances to engulf lipids and be converted to foam cells similarly to macrophages (61, 62). The increased lipid catabolism observed both in human and murine MoDCs could in turn contribute to plaque instability (60, 63). This latter hypothesis is supported by functional studies on human MoDCs treated with oxidized low density lipoproteins (oxLDL) which resulted in increased DCs phagocytic capacity and subsequent naïve T cells priming (64). However, it should be noted that foam cells derived mostly from anti-inflammatory macrophages (M2 subset) (65) and express many lipid-processing and low levels of inflammatory genes resulting in the activation of anti-inflammatory and pro-fibrotic pathways compared to non-foam macrophages (66, 67). While a conclusive effect of foam macrophages in atherosclerosis is still debated, we can speculate that foamy MoDCs might present a similar phenotype, but further studies are warranted to test their contribution to disease progression.

In summary, although several studies have investigated the role of different DC subsets in the context of atherosclerosis a non-univocal function was observed (Table 2). This could be the consequence of the specific context and the type of antigens presented by DCs, which could then differently polarize T lymphocytes in a more activated or in a more suppressive subset as detailed below.

## T cells in atherosclerosis

During their life, T cells acquire different functional states associated to a specific role and localization within the body. When not activated, T cells are usually referred as naïve T cells, and mainly circulate in the bloodstream or are confined in lymphoid tissues, as lymph nodes, from where, following activation, they migrate to distal and inflamed tissues to exert an effector response. T cells are mainly divided in CD4^+^ and CD8^+^ T cells which differ in their response to stimulation. CD4^+^ or helper T cells are classified in different subclasses (Th1, Th2, Th17, and Tregs are among the most studied and described subsets) depending on the cytokines produced after priming by antigen presenting cells; instead, CD8^+^ T cells are classified as a cytotoxic subset given their ability to directly recognize infected or damaged cells and mediate their killing. After the initial stimulation, a pull of memory T cells persists and contributes to the immunological memory to guarantee a rapid response in case of a second encounter with the same antigen; this leads to the rise in memory cells with age.

### Effector T cells

Patients with cardiovascular disease have increased levels of memory CD4^+^ T cells with an effector phenotype (TEM, T effector memory) compared to age and sex matched healthy controls; this finding is paralleled by the positive correlation of circulating TEM levels with the extent of the atherosclerotic plaque in experimental models, or with the severity of atherosclerosis associated CV events in humans (68). More recently, single-cell proteomic and transcriptomic analysis of atherosclerotic plaques from patients with symptomatic cerebrovascular diseases showed the presence of increased TEM levels as compared to plaques from asymptomatic patients (69). Although these approaches have demonstrated that T cells represent approximately 50% of the leukocytes localized in advanced atherosclerotic plaques (70, 71), it has to be taken into consideration the possibility that technical issues during tissue processing—leading to a differential cellular damage—might favor the preservation of T cells over other immune cell subsets (70, 71) and thus provide a still incomplete description of leukocyte subsets distribution in advanced plaque. Besides, compared to T cells landscape in peripheral blood, a more differentiated, activated, and at least in part, (72) exhausted T cell phenotype has been detected, particularly in the fibrous cap (73, 74), or in the adventitia of aged lesions (75, 76). Indeed, the presence of atherosclerosis-associated lymphoid aggregates named ATLOs (arterial tertiary lymphoid organs) has been documented within the adventitia of large and medium sized arteries (77) and by taking advantage from experimental models, the accumulation of leukocytes (including T cells) has been documented with disease progression showing a positive correlation between adventitial infiltrates and atherosclerotic burden (78). ATLOs are involved in antigen-specific primary T cell response, as in this site, naive T cells are first recruited and then maturate to memory cells—both effector, TEM, and central, TCM -, or induced to Tregs (79). Studies using specific transgenic mice, where the structure and the cellularity of tertiary lymphoid organs is disrupted, show that ATLOs may have an anti-atherogenic effect (79). By contrast, antigen presentation by DCs to CD4^+^ T cells in the arterial wall causes local T cell activation and production of proinflammatory cytokines, contributing to atherosclerosis (80), and this event has been recently demonstrated to involved also the direct priming of naïve T cells in the aorta (81).

The contribution of T cell subsets to atherosclerosis has been investigated using several different approaches and contrasting results were observed. Mice models lacking lymphocytes (athymic *nu/nu* mice—deficient in B and T cells and ILCs), or *Rag1^–/–^* mice (deficient in B and T cells) (72, 82–87) or the use of T cell depletion models were shown either to be protected from atherosclerosis or to develop more atherosclerosis (72, 84, 88, 89). The reason for these differences could rely on the type of atherosclerosis model used and the timing of the experiments. In general, a pathogenic role of T cells has been confirmed at early stages of the disease (72), while their causal contribution during the advanced stages has been questioned in hypercholesterolemic *Apoe^–/–^* mice (82, 83). Nevertheless, several works have depicted the involvement of T cells in the different stages of the disease, including the initiation, progression, and regression, till to atherosclerotic plaques rupture or erosion, suggesting that their activation and polarization toward effector subsets sustain the pathological immune-inflammatory response (70). However, while CD4^+^ T cells activation was shown to be essentially proatherogenic (72), the contribution of CD8^+^ T cells still appears more controversial (90).

To note, the pro-inflammatory polarization of CD4^+^ T cells has been shown to depend on the type of co-stimulatory signals and cytokines provided by antigen presenting cells, including local macrophages or B cells in the adventitia. Among the main CD4^+^ T helper (Th) subsets, Th1—characterized by the expression of T-box transcription factor TBX21 (T-bet), the chemokine receptors CXCR3 and CCR5 and the release of IFNγ—are the most prominent subsets in the lesion and have been extensively proved to be atherogenic (91); by contrast, more controversial is the contribution of Th2—expressing the transcription factor GATA3 and mainly producing IL-4, IL-5, IL-10, and IL-13 cytokines—and Th17—characterized by the expression of the transcription factor nuclear receptor RORγt and the production of IL-17, IL-6, GM-CSF, or IL-10—where both pro- or anti- and even null effects on atherosclerosis have been reported [extensively reviewed by Saigusa et al. (70)].

Despite these discrepancies in T helper contribution to the disease, the adoptive transfer of CD4^+^ T cells has been shown to aggravate atherosclerosis in immunodeficient *Apoe^–/–^* mice (70, 92–95), while the transfer of other specific subsets, such as the immunosuppressive Tregs, limits plaque progression.

### Immunosuppressive T regulatory cells

Tregs are immunosuppressive cells originating from the thymus following a two-step process: first, TCR stimulation on immature CD4^+^ single positive thymocytes leads to the generation of CD25^+^FoxP3^–^ Treg cell progenitors (TregPs), then these precursors are converted into mature Tregs via the upregulation of FoxP3 in a cytokine-dependent manner, mainly involving IL-2, IL-15, and IL-7 (96). While this subset is known as natural (nTreg), Treg may be induced also in the periphery from CD4^+^ Foxp3^–^ conventional T cells in the presence of specific antigens/low dose antigens and suboptimal co-stimulation, but also under chronic inflammation (97)—peripheral Treg (pTreg)—or generated *in vitro* in the presence of TGF-β and IL-2 (98, 99), inducible Treg (iTreg). There are still no suitable surface markers to distinguish nTreg from pTreg, despite the intracellular transcription factor Helios (100) has been shown to be particularly expressed by nTreg and the specific mechanisms used by these subsets to control inflammation remain largely unresolved. By recognizing specific self-antigens and inhibiting autoreactive T lymphocytes to differentiate into T helper cells (101), and releasing immunosuppressive cytokines, such as IL-10, TGF-β, and IL-35, Tregs control autoimmune responses (102, 103) and the hyperactivation of immune system. The immunosuppressive effect of Tregs extents also to the modulation of antigen presentation by DCs. In this case the interaction of CTLA-4, an immune checkpoint expressed by Tregs, with co-stimulatory CD80/CD86 receptors on DCs, reduces the expression of these DC receptors, and increases IDO-1 expression which leads to effector T cell starvation, due to increased tryptophan catabolism (104). In line with this, Tregs are able to influence the organization of the immunological synapse, thus impairing DCs ability to recognize and therefore present antigens. In the context of atherosclerosis, Tregs play a protective role by dampening effector T cells, promoting pro-resolving type 2 macrophage phenotype (M2) and suppress foam cell formation within the lesion (101); indeed, many strategies aimed at depleting Tregs, resulted in increased atherosclerosis (84, 105), while those improving Treg levels (106) or enhancing their localization in the atherosclerotic plaque (54) were shown to ameliorate atherosclerosis. However, during the progression of the disease, Treg levels decline in the circulation and within the atherosclerotic plaque of experimental mice, in favor of an increase in CD4^+^ effector T cells. To note, more than half of CD4^+^ T cells reactive to ApoB peptides express FoxP3 in individuals without cardiovascular disease but dramatically decrease and acquire the expression of RORγt and T-bet transcription factors in patients with subclinical atherosclerosis (107). This pathological conversion from immunosuppressive toward effector Treg contributes to disease progression and clearly highlight the intricate role of T cell subsets reflecting their different plasticity and activation during atherosclerosis (Table 3).

**TABLE 3:** Effector T cells and immunosuppressive T regulatory cells-related studies in atherosclerosis.

	Atherosclerosis mouse model	T mouse model	Effects on immune response	Effects on atherosclerosis
Effector T cells	20 weeks atherogenic diet	Anti-CD4 and/or anti-CD8 Ab injection	T cells ablation	Atheroprotective at early stages (72)
	31–32 weeks atherogenic diet	Athymic *nu/nu* mice)	Mature T cells deficiency	Atheroprotective (72)
	*Apoe^–/–^* mice	*Rag1^–/–^* mice	Mature T cells deficiency	Atheroprotective (82) Minor role in atherosclerosis (82)
		*Rag2^–/–^* mice	Mature T cells deficiency	Atheroprotective but controversial (85)
		*CD4^–/–^* mice	CD4^+^ depletion	Atheroprotective (86)
	*Ldlr^–/–^*	*Rag1^–/–^* mice	Mature T cells deficiency	Proatherogenic (83)
	BALB/c*-Ldlr^–/–^*	*Rag1^–/–^* mice	Severe B- and T-cells immunodeficiency	Proatherogenic (87)
		*Rag1^–/–^ Il2rg^–/–^* mice	Severe B-, T- and NK cells immunodeficiency	Proatherogenic (87)
	*Apoe^–/–^* mice	CD4^+^ transfer to *scid/scid* mice	Immunodeficiency	Proatherogenic (92)
	*Apoe^–/–^* mice	CD4^+^ transfer from LDL-immunized mice to *scid/scid* mice	Immunodeficiency	Proatherogenic (93)
Immunosuppressive T regulatory cells	Irradiated *Ldlr^–/–^*	CD80/CD86^–/–^ or CD28^–/–^ BM cells injection	Altered Tregs generation (reduced Treg levels)	Proatherogenic (84)
	Irradiated *Ldlr^–/–^*	DEREG (depletion of regulatory T cells) mice BM	Tregs depletion	Proatherogenic (105)
	*Apoe^–/–^* mice	Isolated Tregs transfer	Improving Tregs levels	Atheroprotective (106)
	*Ldlr^–/–^*	Engineered Tregs transfer	No systemic effect, plaque anti-inflammatory phenotype	Atheroprotective (54)
		Anti-CD3 antibody (CD3-Ab) injection	Reduced CD4^+^ T cells and increased the Tregs proportion	Atheroprotective (88)
		Anti-CD3 and anti-CD25 Abs injection	Reduced CD4^+^ T cells and Tregs	Atheroprotective role of Tregs (88)
		anti-CD8α Ab	CD8^+^ T cells depletion	Atheroprotective (89)

## Loss of peripheral tolerance in the context of atherosclerosis

As described above, the activation of the adaptive immune response requires the recognition of “not-self” epitopes presented by MHC class I and II complexes—expressed on all nucleated cells or on antigen presenting cells, respectively, by CD4^+^ and CD8^+^ T cells. In parallel the incorrect activation of immune system toward self-antigens in steady state conditions is supervised by tolerogenic DCs which concur to the negative selection of autoreactive T cells within the thymus as well as to the modulation of self-reactive T cell anergy and Treg expansion in tissues (108).

Commonly, immature DCs in the periphery exert a tolerant role because of the low expression of co-stimulatory molecules and cytokines production (109). The shift from a tolerogenic to a pro-inflammatory phenotype is influenced by antigen uptake (mediated or not by toll-like receptors) and by the local milieu (enriched or not in pro-inflammatory cytokines) (108). Indeed, the recognition of self-antigens in a non-inflamed environment leads to the anergy of autoreactive lymphocytes due to the absence of costimulatory molecules during DCs-mediated antigen presentation and is paralleled by Treg activation, induced through IL-2, TGF-β, and IL-10 production (110). This function should be maintained also during inflammation to preserve tolerance to self-antigens released by damaged tissues. Vice versa, under chronic inflammatory conditions antigens are captured mainly in a TLR-dependent manner, and this promotes the maturation of DCs as well as the differentiation of monocytes in MoDCs which, together, could trigger the activation of T cells also against self- or modified self-antigens (8, 111) mainly generated by apoptotic cells. To limit this possibility, a proper clearance of apoptotic cells via phagocytes was proposed to play a key role in reducing the amount of self-antigens potentially driving auto-immune responses under these conditions (112).

The latter mechanism has been proposed to be critical also during atherosclerosis (110, 113) and, when impaired, could favor the exposure of potential atherosclerosis-associated antigens. Indeed, antigen-specific T cell clones have been detected within the atherosclerotic plaque of mice and humans (114, 115). These findings clearly highlight that a “break of tolerance” could occur during atherosclerosis. The first self-antigen proposed to be involved in this process was ApoB, suggesting the possibility that atherosclerosis could be the consequence of an auto-immune response (116). Later on, additional antigen reactive T cell clones were identified in atherosclerotic plaques, including those directed toward oxLDL, or HSPs (heat shock proteins), but also directed toward not-self antigens such as pathogens like Cytomegalovirus (CMV), hepatitis C virus (HCV), HIV, human papillomavirus (HPV), and others (107, 117–126).

## Targeting dendritic cell-T cell axis in atherosclerosis

The increasing understanding of the role of immunity in atherosclerosis is providing new options for the treatment of cardiovascular disease on top of the control of classical risk factors, such as dyslipidemia (127–130). Indeed, the identification of specific atherosclerosis-associated antigens suggests the possibility to boost atheroprotective immune responses (131) by targeting the crosstalk between DCs and T cells to train immunity toward a tolerogenic response.

Pioneering studies by Pakinski and Ameli on hypercholesterolemic rabbits showed the atheroprotective effect of immunization with modified LDL; this, in turn, fueled the use of ApoB peptides, that are specifically MHC II restricted, to trigger a CD4^+^ T cell response (132) and Treg increase (131), as a vaccination strategy in experimental atherosclerosis (132–135). Despite some MHC-I epitopes against murine and human ApoB have also been identified, the largest effort has been devoted in identifying MHC-II-restricted epitopes of ApoB (3), that could evoke a robust and atheroprotective response. Indeed, the identification of the epitopes does not predict how strong the immune response to these peptides will be (3); for example, anti-human ApoB100 specific CD8^+^ T cells in humanized mice did not result in atheroprotection (136), in contrast to CD4^+^ T cells reactive to ApoB peptides that, through a detailed phenotypic and transcriptomic analyses of MHC class II -restricted, antigen-specific T cells, were shown to present a pro-inflammatory signature during advanced phases of atherosclerosis (107, 137). This suggests that the immune subset involved and the timing of inducing the response toward ApoB could differently affect disease progression.

Several efforts have been done to identify the best antigen/antigenic epitope to mount a tolerogenic response in atherosclerosis (138). DCs pulsed *in vitro* with oxLDL (139) or with ApoB100 (140) and then injected in hypercholesterolemic mice have been shown to reduce atherosclerosis development. On the contrary, DCs pulsed with malondialdehyde modified LDL (MDA-LDL-DCs) and injected in *ApoE^–/–^ mice* increased atherosclerosis (141). These different findings could, however, depend on the protocol used to pulse DCs, including the type of adjuvant utilized (139, 141). Usually DNA sequences containing unmethylated cytosine guanine dinucleotides (CpG) motifs are used (142), while specific adjuvants, as Alum and Freund’s incomplete adjuvant, were shown to promote the switch toward a Th2 rather than a Th1 response (138). Additional strategies to improve DC-based approaches could take into account the possibility of administering free antigens, or fusion proteins which allow the specific targeting of DCs subsets (143). The generation of *in vitro* tolerogenic-DCs culture by maintaining a lower expression of co-stimulatory molecules to promote Treg skewing (101), together with the possibility of silencing co-stimulatory molecules or the NF-KB pathway (144), are under investigation in different auto-immune diseases, including type 1 diabetes treatment and rheumatoid arthritis (RA).

Together with the improvement of DC tolerogenic function, the possibility of targeting Tregs, to exploit their atheroprotective functions (110), is under intense investigation. Initial reports in humans showed that the treatment with anti-CD3 monoclonal antibody could restore tolerance in type 1 diabetes by inducing Treg response (145); on this premise, oral administration of anti-CD3-based therapy was shown to improve TGF-β production, Treg expansion and ameliorate atherosclerosis (146). Increased TGF-β production and levels of Foxp3^+^ cells were also observed in atheroprone mice following oral administration of oxLDL (147). In parallel, Treg supplementation was also shown to improve atherosclerosis by increasing phagocytosis of apoptotic debris thus contributing to the maintenance of immune balance (110). Other factors that have been reported to increase Tregs were granulocyte-colony-stimulating factor (G-CSF) by the modulation of DC and T cell functions, and rapamycin, the inhibitor of mTOR, which promotes Treg expansion (146). More recently, the effect of low doses of IL-2 (that is crucial for Treg homeostasis) on CVD in humans is currently being tested in the LILACS clinical trial (146). Interestingly, also oral administration of specific molecules such as D3 (calcitriol) was shown to promote the induction of tolerogenic DCs and Tregs (109). Moreover the possibility of forcing DC migration from the gut to the aorta following oral administration of multi-antigenic structures appears promising in decreasing CD11c^+^ cells and increasing Tregs in the plaque (148).

An attracting strategy to maintain immune tolerance toward self-antigens during atherosclerosis could be represented by the modulation of inhibitory pathways, including PD-1/PD-L1 and CTLA-4 receptors (149, 150). These immune checkpoint proteins expressed by APCs and T cells are involved in controlling T cell activation, cytokines production and the interaction between immune cells and non-immune cells, such as endothelial cells. The inhibition of CD80 and CD86 with abatacept limited atherogenesis and similar results have been observed with CTLA-4 stimulation (151, 152), while approaches blocking PD-1/PD-L1 or 2 pathways aggravated atherosclerosis (153–156). Obviously these strategies hold the limitation that the activation of immune checkpoint proteins could lead to severe immune suppression (149), limiting the number of subjects where this approach could be beneficial without promoting harms.

## Concluding remarks

Atherosclerosis results from the combination of lipid accumulation and unbalanced immune response. Lot of attention has been placed on addressing the “break-of-tolerance” hypothesis where T cells react against self-antigen associated to cardiovascular risk factors (such as ApoB peptides and modified LDL). Of note, T cell activation is orchestrated by antigen presentation by professional APCs in a pro- or anti-inflammatory microenvironment. In the last few years, DCs have emerged as a key antigen presenting cell subset involved in cardiovascular inflammation and it is becoming clear that the recognition of antigens by T cells and their activation is primed by phenotypical and functionally different DC subsets. These observations pave the way toward a better understanding of DC subset development, polarization and T cell training in atherosclerosis. The growing interest in using DC-based therapies (e.g., in cancer and autoimmune diseases) encourages to deepen the understanding of the role of DCs—and their subsets—within the atherosclerotic plaque. The possibility of targeting specific DC functions would offer an innovative pharmacological strategy to modulate the immune-inflammatory response in the context of cardiovascular diseases.

## Author contributions

RB and FB drafted and wrote the manuscript. GN drafted and edited the manuscript. All authors reviewed the manuscript and approved the submitted version.
